# Lymphangitic Carcinomatosis Presenting as Cough: A Case of Occult Metastatic Prostate Adenocarcinoma

**DOI:** 10.1155/2024/9915126

**Published:** 2024-11-15

**Authors:** Sharanya Kumar, Allen Seylani, Rahul Tuli, Eric Abedi, Keerti Khandelwal

**Affiliations:** ^1^Hematology/Oncology, Riverside University Health System, Moreno Valley, California, USA; ^2^University of California Riverside School of Medicine, Riverside, California, USA

**Keywords:** lymphangitic carcinomatosis, metastatic, PET-CT, prostate cancer

## Abstract

**Background:** Lymphangitic carcinomatosis (LC), a hallmark of advanced metastatic cancer with a poor prognosis, primarily impacts the lymphatic system of the lungs, manifesting as progressive breathlessness, cough, or hemoptysis. While prostate cancer commonly metastasizes to bones and regional lymph nodes, lung involvement is rare. This case features a patient in generally good health who presented with an insidious dry cough, leading to a diagnosis of Stage 4 prostatic adenocarcinoma with rare lymphangitic spread to the lungs.

**Case Presentation:** A 70-year-old male in good health presented with chest tightness, a dry cough, and sudden left testicular swelling. Imaging revealed interstitial lung markings, severe left hydronephrosis, and prostatomegaly. A prostate biopsy confirmed adenocarcinoma. A PET-CT scan raised significant concern for LC, prompting the initiation of urgent inpatient chemotherapy with docetaxel.

**Conclusion:** LC is a metastatic pattern commonly associated with solid tumors, particularly breast, gastric, and lung cancers. Its occurrence in prostate cancer is exceptionally rare. This condition is typically linked with advanced disease and a poor prognosis, often serving as a critical indicator of an underlying malignancy that may otherwise go undetected.

## 1. Background

Lymphangitic carcinomatosis (LC) is a severe, late-stage manifestation of metastatic cancer that predominantly affects the pulmonary lymphatic system. It is a hallmark of advanced disease and carries a very poor prognosis, with most patients having a survival expectancy of less than 3 months after diagnosis [1]. The pathophysiology involves the infiltration of malignant cells into the lymphatic channels, leading to their obstruction and subsequent interstitial thickening, inflammation, and eventual fibrosis [2]. This process can result in progressive respiratory distress, with dyspnea being the most common presenting symptom, though cough and hemoptysis can also occur [3]. Radiographically, LC can be challenging to diagnose, as it often mimics other interstitial lung diseases (ILDs), such as pulmonary fibrosis [4]. While LC is most commonly associated with breast, gastric, and lung cancers it is notably rare in prostate cancer. In prostate cancer, metastatic spread typically occurs via lymphatic or hematogenous routes, with the former representing the majority of cases [5]. Lymphatic spread in metastatic prostate cancer often travels to bone and regional lymph nodes in the retroperitoneum and pelvis, with lung and pleural involvement rarely seen, making lymphangitic spread to the lungs in prostate cancer an unusual and noteworthy occurrence [6]. In this case, we present a patient who was otherwise in good health but developed an insidious dry cough. Initial imaging revealed diffuse reticular opacities, which prompted further evaluation and ultimately led to the diagnosis of Stage 4 prostatic adenocarcinoma with lymphangitic spread to the lungs.

## 2. Case Presentation

**Figure 1:**
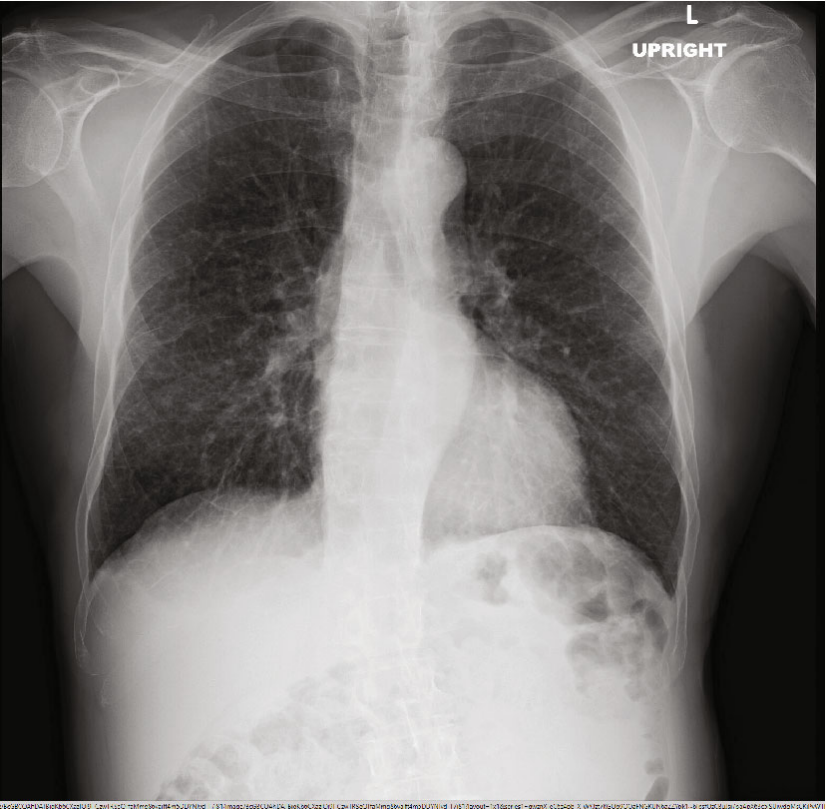
Chest X-ray demonstrating scattered reticulations, suggestive of interstitial lung disease, with nodular opacities noted in the right midzone.

A 70-year-old active male with a history of hypertension presented to the hospital with chest tightness, a dry cough persisting for 1 month, and sudden, painless left testicular swelling. Vitals and laboratory results were unremarkable. An initial chest X-ray revealed scattered coarse interstitial markings with bronchial thickening and subpleural reticulations (Figure 1). A testicular ultrasound showed heterogeneous parenchyma in the left testicle. The testicular swelling resolved spontaneously within 3 weeks, but the cough persisted.

**Figure 2:**
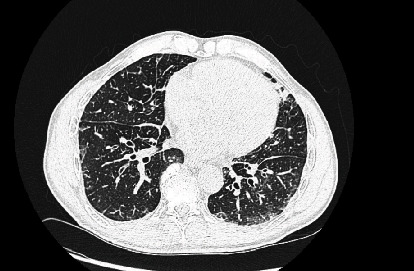
CT chest without contrast showing interlobular septal thickening, bronchiectasis, and basilar predominant lung opacities.

During outpatient follow-up, a chest CT revealed interlobular septal thickening, bronchiectasis, and basilar predominant lung opacities suggestive of ILD, but also identified severe left hydronephrosis (Figure 2). A subsequent CT of the abdomen and pelvis showed an irregular bladder contour with significant prostatomegaly. A referral to urology led to a digital rectal exam (DRE) that revealed a firm and enlarged prostate. His PSA level was elevated at 220.4 ng/mL, and a prostate biopsy confirmed adenocarcinoma with a Gleason score of 9 (4 + 5). Bicalutamide 50 mg daily was initiated, with plans to start Lupron within 2–3 weeks.

**Figure 3:**
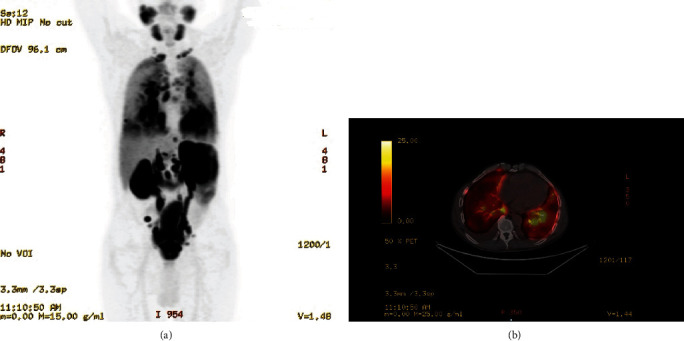
(a) Whole-body PET scan showing extensive hypermetabolic activity throughout the pulmonary parenchyma, with discrete nodularity in the lingula, highly suggestive of diffuse lymphangitic carcinomatosis. Additionally, there is a significant radiotracer uptake in the prostate gland as well as mediastinal, supraclavicular, and pelvic lymph nodes. (b) Axial PET-CT image demonstrating significant fluorodeoxyglucose (FDG) uptake within the beaded interlobular septal thickening of the upper lobes, as well as throughout both lungs, extending into the perihilar regions and mediastinal lymph nodes.

An urgent PET-CT scan revealed diffuse hypermetabolic activity in the bilateral pulmonary parenchyma, raising concerns for LC, and identified a small nodule in the lingula (Figures 3(a) and 3(b)). Metastatic disease was also detected in the supraclavicular and mediastinal lymph nodes, right posterior iliac spine, and pelvic lymph nodes. Due to the advanced nature of the prostate cancer, the patient was admitted for urgent inpatient chemotherapy following the PET-CT results. The pulmonary metastases were approximately 5 mm in size, rendering them unsuitable for biopsy by Interventional Radiology. Given the aggressive nature of the disease, a triple therapy regimen was planned, including pretreatment with bicalutamide, followed by Lupron, docetaxel, and darolutamide. Next-generation sequencing (NGS) indicated BRCA negativity and androgen receptor (AR) positivity. The follow-up plan includes monitoring PSA levels and tracking overall symptom improvement.

Following treatment with bicalutamide, a single Lupron injection, three cycles of docetaxel, and one dose of darolutamide, the patient showed significant improvement in breathing and was able to walk longer distances. His PSA level also dropped from over 220 to 1.0 ng/mL.

## 3. Discussion

Prostate cancer is the second most common cancer globally and the leading cancer among men in the United States. The overall death rate from prostate cancer across all stages is approximately 0.019 per 100,000 men [7]. Metastatic prostate cancer has a 5-year survival rate of approximately 30%, significantly lower than the nearly 97% survival rate for localized or regional disease. The most common sites of metastasis are the bones, lymph nodes, and liver. Lung metastasis is rare, often identified only in postmortem studies [8].

Lymphangitic spread of cancer is a grave prognostic indicator, with a median survival of just 2 months from the onset of pulmonary symptoms to death. This rapid decline underscores the aggressive nature of LC and its association with advanced, often terminal, stages of cancer [9]. While diagnosis is typically established through transbronchial biopsy, in cases where patients have compromised respiratory status, it is often made using a combination of clinical and radiographic findings. In patients with prostate cancer, significant improvements have been observed with total androgen blockade [10]. Therefore, timely diagnosis of lymphangitic spread upon clinical suspicion is crucial for initiating prompt treatment.

Prostate cancer is challenging to diagnose due to its often asymptomatic nature. Its indolent progression means it frequently remains undetected until metastatic spread leads to symptoms, such as urinary retention from urethral compression or bone pain from osteoblastic metastases [11]. LC represents severe cases of metastatic spread and is an uncommon pattern of metastasis in prostate cancer cases. It presents a diagnostic difficulty due to its ability to mimic many primary respiratory disorders, namely, ILD [12]. Patients typically have symptoms such as progressive dyspnea on exertion, cough, and rarely hemoptysis. In patients without significant pulmonary risk factors, such as smoking history, who develop slowly progressive respiratory symptoms and radiographic findings suggestive of ILD, it is essential to consider lymphangitic spread as a potential cause and conduct further investigation into the underlying etiology [13]. ILD generally follows a chronic, progressive course, whereas LC leads to a more rapid decline in respiratory function. However, differentiating between them is clinically and radiographically challenging. Therefore, additional information through thorough history-taking or diagnostic modalities is necessary to identify a primary malignancy.

Although the occurrence of LC cannot be predicted, it should be a key differential diagnosis, particularly in patients who have not undergone age-appropriate cancer screening. For prostate cancer, routine PSA testing remains controversial due to various confounding factors, including medications, age, and infection [14]. Additionally, DREs have shown limited sensitivity and specificity in detecting early cases of prostate cancer [15]. Alternatively, they are unlikely to assist in early diagnosis during screening, as they are more apt to detect abnormalities in patients with already widespread disease. These tests can be performed if all other causes of respiratory symptoms have been excluded and there remains concern about a potential malignancy.

## Data Availability

Data sharing is not applicable to this article as no new data were created or analyzed in this study.
